# Left lower extremity venogram and common iliac venoplasty: guidewire-induced arrhythmia

**DOI:** 10.1097/MS9.0000000000003094

**Published:** 2025-03-18

**Authors:** Vladislav Pavlovich Zhitny, Brett Dixon, Ryan Jannoud, Aaron N. Primm, Kenny Do, Eric Kawana, Benjamin Vachirakorntong, Jenifer Do, Anke Wang, Jean-Paul Garre, Leroy Phillips

**Affiliations:** a Department of Anesthesiology, Perioperative Care, and Pain Medicine, New York University, New York City, NY, USA; b Kirk Kerkorian School of Medicine, University of Nevada, Las Vegas, Las Vegas, NV, USA; c Touro University Nevada College of Osteopathic Medicine, Henderson, NV, USA; d School of Life Sciences, University of Nevada, Las Vegas, Las Vegas, NV, USA

**Keywords:** heart block, left bundle branch block, pulmonary artery catheter, right bundle branch block

## Abstract

**Introduction and importance:**

Modern interventional procedures utilizing guidewires for venous and arterial access, while generally safe, can occasionally lead to complications that challenge the skills of anesthesia providers.

**Case presentation:**

We describe a case in which an angioplasty guidewire migrated from the left common iliac vein into the main pulmonary artery, resulting in premature ventricular contractions (PVCs). During the procedure, intraoperative electrocardiogram (ECG) monitoring detected multiple PVCs that persisted without spontaneous resolution. Fluoroscopy subsequently revealed the guidewire’s migration into the pulmonary artery.

**Clinical discussion:**

The wire was successfully withdrawn under live imaging, which resolved the PVCs without further complications. Guidewire misplacement in venous access procedures is uncommon but can cause mechanical irritation to cardiac tissue, leading to arrhythmias such as PVCs. In this case, timely recognition and intervention prevented further escalation of the arrhythmia.

**Conclusion:**

This case underscores the potential for guidewire migration to cause significant intraoperative complications, such as arrhythmias, and emphasizes the importance of vigilant ECG and hemodynamic monitoring during procedures involving guidewires, even those performed on the lower extremities.

## Introduction

Guidewire-induced arrhythmias are a significant concern during venous and arterial catheterization, as they can potentially progress to malignant arrhythmias^[1]^. While transient arrhythmias are a known complication of intrathoracic procedures, such as central venous access, ectopic arrhythmias caused by a guidewire remain rare and under-documented^[1]^. One such arrhythmia is right bundle branch block (RBBB), which can occur when the guidewire inadvertently stimulates the right bundle branch during catheter insertion^[2,3]^. This is particularly problematic in patients with preexisting left bundle branch block (LBBB), as it increases the risk of complete heart block^[4,5]^.

**HIGHLIGHTS**

- Rare case of guidewire-induced arrhythmia: This report details a rare but significant occurrence of guidewire-induced arrhythmia during a left lower extremity venogram and iliac venoplasty procedure. The guidewire migrated from the left common iliac vein into the pulmonary artery, causing premature ventricular contractions (PVCs).
- Importance of vigilant monitoring: The case underscores the critical need for close intraoperative monitoring, especially electrocardiogram and hemodynamic parameters, during procedures involving guidewires, even those typically considered lower risk, such as lower extremity interventions.
- Successful management of arrhythmia: The arrhythmia resolved once the guidewire was carefully withdrawn under live fluoroscopic guidance, demonstrating an effective management strategy for this complication without further intervention or adverse events.
- Implications for anesthesia providers: This report serves as a valuable reference for anesthesia providers and other healthcare professionals, emphasizing the potential for unexpected complications during venous interventions and offering practical management guidance.
- Clinical significance for patients with preexisting cardiac conditions: Patients with preexisting conditions, such as left bundle branch block, are at increased risk of complications during invasive procedures. This case highlights the need for special consideration and preparedness, including the availability of pacing equipment in such scenarios.
- Educational value and awareness: The case provides valuable teaching points for clinicians, raising awareness of guidewire migration as a possible cause of intraoperative arrhythmias and offering insight into preventive measures and responsive actions during similar procedures.

Premature ventricular contractions (PVCs) can be observed during a myriad of procedures known to induce aberrant conduction. These procedures include, but are not limited to, upper extremity central line placement, pulmonary artery catheter (PAC) placement, hemodialysis catheter placement, percutaneous coronary intervention, annuloplasty, valvuloplasty, transcatheter aortic valve replacement, cardiac surgery, and neurointerventional radiology procedures^[6]^.

In this report, we present a case of an angioplasty guidewire inducing PVCs by migrating from the left common iliac vein into the right ventricle and subsequently into the pulmonary artery. This case underscores the importance of careful monitoring during catheter placement and explores strategies to manage these rare but serious conditions. While relatively rare, there have been documented cases of PAC guidewires causing complications, highlighting the broader clinical relevance of this phenomenon^[7]^. We aim to improve awareness and provide guidance on minimizing risks associated with guidewire-induced arrhythmias.

Written consent was obtained from the patient to write this case report.

## Case presentation

A 48-year-old male with a past medical history of gastroesophageal reflux disease, metabolic syndrome, venous insufficiency of the lower left extremity (LLE) with chronic venous stasis, previous deep vein thrombosis (DVT) in the LLE (status post apixaban treatment), and vitamin D deficiency presented with chronic left lower venous insufficiency causing ulceration. He previously underwent venous ablation and now presents for LLE venogram with intravascular ultrasound and left common iliac venoplasty.

On physical examination, the patient was classified as Mallampati II. His cardiovascular, pulmonary, neurological, and abdominal examinations were normal. He had a body mass index of 33.73 and a STOP-BANG score of 1 (male sex). Examination of the patient’s extremities revealed evidence of venous insufficiency, causing ulceration and discoloration of the left calf.

**Figure 1.**
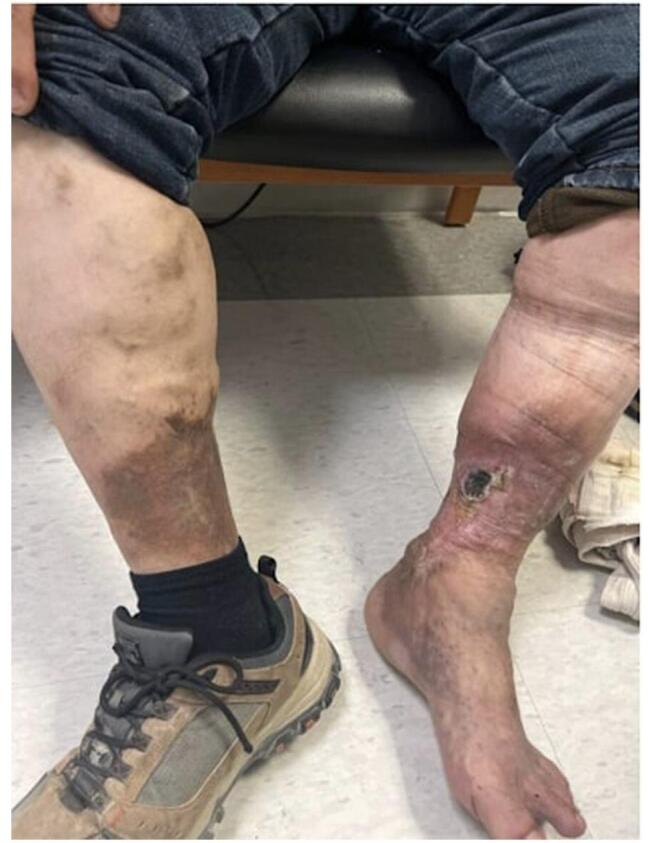
Physical examination showing left leg medial ulcer and chronic venous stasis.

Palpable dorsalis pedis pulses were noted bilaterally (Fig. 1).

The patient reported a metabolic equivalent of tasks greater than 4, indicating his ability to walk 10 blocks and climb 8 flights of stairs at a moderate pace without experiencing chest pain or shortness of breath. He works as a plumber and has a past surgical history of varicose vein surgery, small bowel obstruction surgery, and Roux-en-Y gastric sleeve laparoscopy.

**Figure 2.**
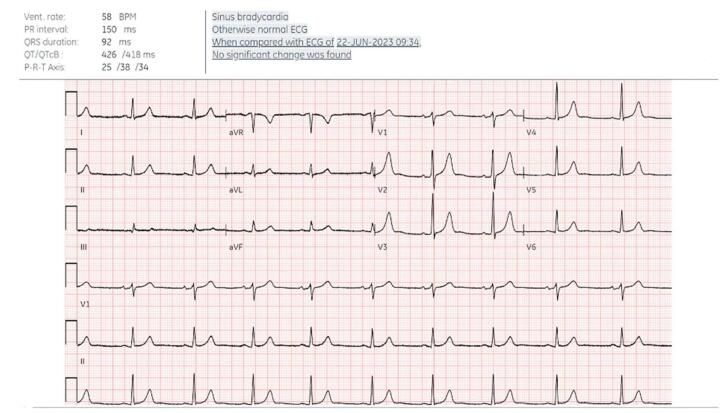
Preoperative testing sinus bradycardia by ECG.

His current medications include ergocalciferol 50 000 IU and multivitamins. Laboratory results showed a hemoglobin level of 13.0 g/dL, platelets at 288 × 10^9^/L, and white blood cells at 5.74 × 10^9^/L. The basic metabolic panel showed sodium at 138 mmol/L, potassium at 4.0 mmol/L, carbon dioxide at 23 mmol/L, creatinine at 0.9 mg/dL, blood urea nitrogen at 22 mg/dL, and glucose at 89 mg/dL. An electrocardiogram (ECG) indicated sinus bradycardia with a ventricular rate of 58 beats per minute and a QT interval of 426 milliseconds (Fig. 2).

A bilateral lower extremity ultrasound revealed a non-occlusive DVT in the left femoral vein, left popliteal vein, and left gastrocnemius vein. Additionally, a bilateral ultrasound of the great saphenous vein and the small saphenous vein showed chronic non-occlusive superficial vein thrombosis (SVT). Finally, the right calf varicose veins demonstrated chronic non-occlusive SVT.

**Figure 3.**
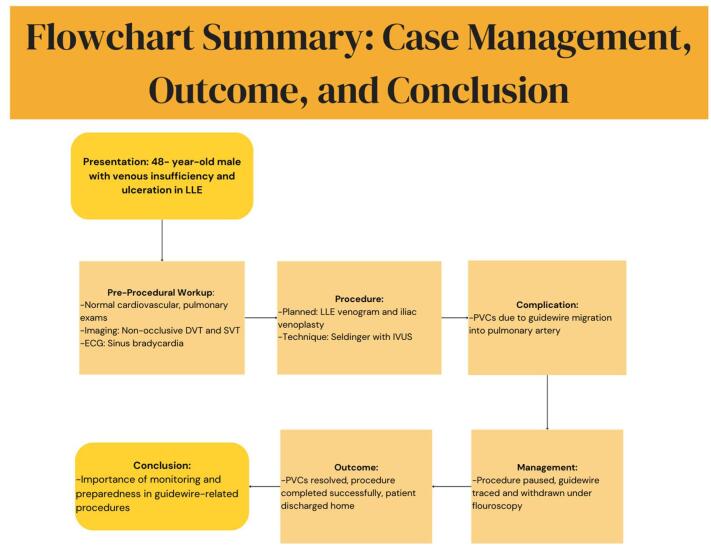
Flowchart summary of case management, outcome, and conclusion.

Deep sedation with propofol and dexmedetomidine infusion was administered. Left femoral access was obtained via the Seldinger technique to investigate for occlusion. A Terumo Glidewire with an angled stiff shaft, measuring 0.035 inches in diameter and 260 cm in length, was used for the procedure. The left common iliac vein was identified via intravascular ultrasound as the culprit lesion and quickly treated. Toward the end of the procedure, PVCs were noted on intraoperative 5-lead ECG monitoring. The patient remained hemodynamically stable during this time. The surgical team was notified, and the procedure was paused. The fluoroscopy imaging did not show the distal end of the guidewire; however, further tracing showed the distal end in a pulmonary artery. The wire was withdrawn under live fluoroscopic guidance, and the PVCs resolved once the wire exited the right atrium. The patient’s vital signs remained stable throughout this time. An emergency crash cart and pacing pads were available in the operating room. The case proceeded without further arrhythmia or complications (Fig. 3). The patient was transferred to the postanesthesia care unit and discharged home uneventfully.

At the 1-month follow-up, the patient presented for the evaluation after undergoing an LLE venogram with venoplasty to the left common iliac vein. The patient reported improvement in both the wound and symptoms. On examination, bilateral dorsalis pedis pulses were palpable, the extremities showed persistent evidence of bilateral hyperpigmentation and varicosities. The left medial gaiter region exhibited a 2 cm × 1 cm healing ulcer with a granulating base and minimal serous damage observed on gauze.

At the 6-month follow-up with internal medicine for an upcoming elective bariatric procedure, cardiovascular screening revealed a blood pressure of 104/57 mmHg, and 107/24 mmHg on recheck. The internal medicine risk stratification pre-procedural clearance showed the patient’s 10-year Atherosclerotic Cardiovascular Disease risk score of 1.7%, calculated per the guidelines by Arnett *et al*^[8]^. No subjective palpitations were noted by the patient during the follow up, no additional ECG screening was ordered by the internal medicine or vascular surgical colleagues.

## Discussion

In this case report, we present the unusual occurrence of an angioplasty guidewire inadvertently migrating into the main pulmonary artery during procedural access, leading to the onset of PVCs. This event introduced a complex medical situation that necessitated immediate mediation and oversight. This case aligns with previous literature describing similar complications that resulted from the displacement of central venous catheter guidewires in other procedures (Table 1)^[3]^.

**Table 1:** Complications commonly seen during placement of pulmonary artery guidewire.

Iatrogenic complications of guidewire placement
Bleeding
Perforation of veins
Hematoma
Thrombosis
Infection
Needle stick injury
Air embolism
Kinking or looping of the wire tip
Breakage of a guidewire
Migration of a guidewire

Our case shares similarities with the routine insertion of PACs and the associated challenges of accessing, inserting, and maintaining catheter position. Arrhythmias are the most common complication during PAC utilization, specifically when the PAC is advanced into the right ventricle and subsequently migrates into the pulmonary artery^[9]^. PVCs or nonsustained ventricular tachycardia are commonly seen and can be resolved by repositioning the catheter^[9]^. Rarely, maintaining the position of the catheter can lead to a pulmonary artery rupture, a life-threatening surgical emergency^[10]^.

While arrhythmias are a well-known complication during PAC utilization, it is important to consider other rhythm disturbances that can occur in the perioperative setting, such as paroxysmal atrioventricular block (AVB). Common causes of AVB include medications such as digitalis and other antiarrhythmic drugs, coronary artery disease, and aging-related degenerative changes in the conduction system. Additional causes include surgery, electrolyte imbalance, endocarditis, tumor, Chagas’ disease, rheumatic heart disease, calcified aortic valve stenosis, myxedema, and inflammatory heart disease are all correlated with AVB^[11]^.

Given these findings, anesthesia providers should closely monitor ECG rhythm and hemodynamic stability during any procedure, particularly those involving intravascular guidewire placement, even in lower extremity interventions. In patients with preexisting LBBB, catheter-induced PVCs can result in a complete heart block, necessitating pacing. Therefore, bedside pacing should be available for all guidewire-related procedures. It is also important to discuss with patients the possibility of a complete heart block in patients who have underlying structural heart disease. Careful assessment and planning are essential to managing risks and ensuring patient safety before any procedure.

One available model for assessment provides exclusion criteria for patients with LBBB undergoing right heart catheterization, who are at risk of developing RBBB or CHB due to right bundle branch trauma^[12]^. In a trial involving 27 consecutive patients with LBBB who developed either RBBB or CHB during the procedure, researchers found that the presence of an r wave greater than or equal to 1 mm in lead V1 suggested intact left-to-right ventricular-septal activation and identified LBBB patients at low risk of CHB during right heart catheterization. These findings suggest that an initial r wave greater than or equal to 1 mm in lead V1, present in approximately 28% of ECGs with classically defined LBBB, may serve as a new exclusion criterion when defining complete LBBB^[12]^.

## Conclusion

This case highlights the rare occurrence of guidewire-induced arrhythmias, particularly in patients with preexisting LBBB. The guidewire’s unexpected migration from the left common iliac vein into the pulmonary artery triggered PVCs, posing a significant risk of progressing to complete heart block. Immediate intervention, including pausing the procedure, using fluoroscopy to trace the guidewire, and carefully withdrawing it, successfully resolved the arrhythmia. Despite this complication, the angioplasty procedure was completed successfully without further adverse events.

This case underscores the importance of close monitoring and preparedness, including having bedside pacing readily available, during all guidewire-related procedures. The guidewire’s migration into the pulmonary artery was likely due to improper directional control during advancement, compounded by patient-specific anatomical factors. Clinicians can mitigate these risks by maintaining optimal visualization with real-time fluoroscopy during the advancement of the guidewire and by careful observation of unusual resistance or movements, and obtaining additional truncal fluoroscopy imaging PVCs are noted on the intra-operative ECGs.

Although no resistance was noted during this procedure, clinicians should carefully assess for any tactile feedback indicating deviation from the intended path of the guidewire. Additionally, the choice of guidewire type may influence the likelihood of unintended migration, and selection should be tailored to the patient’s anatomy and procedure.

This case highlights a vital learning point: routine use of live imaging and preemptive risk stratification can prevent potentially severe complications. Future quality improvement studies should focus on identifying patient-specific and procedural factors contributing to guidewire misdirection, with the goal of developing standardized protocols to enhance safety.

By highlighting this experience, we aim to improve awareness and guide future management of similar high-risk scenarios, ensuring better patient outcomes.

## Data Availability

Data sharing is not applicable to this article.
